# Quasi-cellular systems: stochastic simulation analysis at nanoscale range

**DOI:** 10.1186/1471-2105-14-S7-S7

**Published:** 2013-04-22

**Authors:** Lorenzo Calviello, Pasquale Stano, Fabio Mavelli, Pier Luigi Luisi, Roberto Marangoni

**Affiliations:** 1 Dipartimento di Informatica, Università di Pisa, L.go B. Pontecorvo 3, 56127 Pisa, Italy; 2 Dipartimento di Biologia, Università di Roma III, Via G. Marconi 446, 00146 Roma, Italy; 3 Dipartimento di Chimica, Università di Bari, Via E. Orabona 4, 70121 Bari, Italy; 4 Istituto di Biofisica del CNR, Via G. Moruzzi 1, 56124 Pisa, Italy

## Abstract

**Background:**

The wet-lab synthesis of the simplest forms of life (minimal cells) is a challenging aspect in modern synthetic biology. Quasi-cellular systems able to produce proteins directly from DNA can be obtained by encapsulating the cell-free transcription/translation system PURESYSTEM™(PS) in liposomes. It is possible to detect the intra-vesicle protein production using DNA encoding for GFP and monitoring the fluorescence emission over time. The entrapment of solutes in small-volume liposomes is a fundamental open problem. Stochastic simulation is a valuable tool in the study of biochemical reaction at nanoscale range. QDC (Quick Direct-Method Controlled), a stochastic simulation software based on the well-known Gillespie's SSA algorithm, was used. A suitable model formally describing the PS reactions network was developed, to predict, from inner species concentrations (very difficult to measure in small-volumes), the resulting fluorescence signal (experimentally observable).

**Results:**

Thanks to suitable features specific of QDC, we successfully formalized the dynamical coupling between the transcription and translation processes that occurs in the real PS, thus bypassing the concurrent-only environment of Gillespie's algorithm. Simulations were firstly performed for large liposomes (2.67µm of diameter) entrapping the PS to synthetize GFP. By varying the initial concentrations of the three main classes of molecules involved in the PS (DNA, enzymes, consumables), we were able to stochastically simulate the time-course of GFP-production. The sigmoid fit of the GFP-production curves allowed us to extract three quantitative parameters which are significantly dependent on the various initial states. Then we extended this study for small-volume liposomes (575 nm of diameter), where it is more complex to infer the intra-vesicle composition, due to the expected anomalous entrapment phenomena. We identified almost two extreme states that are forecasted to give rise to significantly different experimental observables.

**Conclusions:**

The present work is the first one describing in the detail the stochastic behavior of the PS. Thanks to our results, an experimental approach is now possible, aimed at recording the GFP production kinetics in very small micro-emulsion droplets or liposomes, and inferring, by using the simulation as a reverse-engineering procedure, the internal solutes distribution, and shed light on the still unknown forces driving the entrapment phenomenon.

## Background

### Toward the construction of synthetic cells

One of the major goals of Synthetic Biology is the *de novo *creation of living organisms in laboratory, an ambitious and challenging goal that promises a profound impact in basic science and in biotechnology [1-3].

The first stage towards this long-term goal is represented by the construction of quasi-cellular systems (also named "minimal cells") that are liposome-based compartment containing the minimal and sufficient number of biomolecules that, by interacting with each other within such a membrane compartment, are able to emulate some of the fundamental properties of living systems.

**Figure 1:**
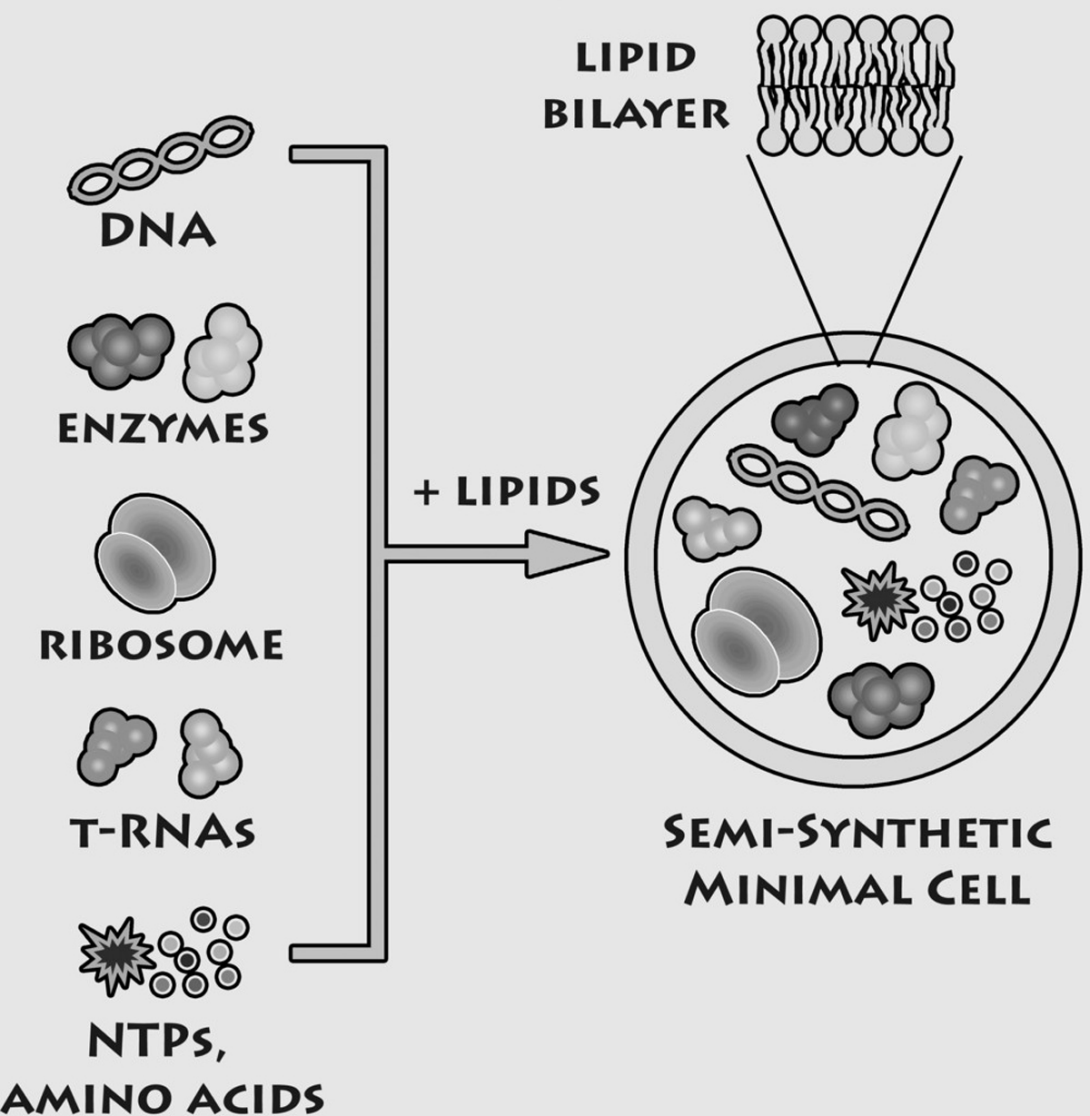
**A Semi-synthetic minimal cell**. Semi-synthetic minimal cells are composed of the minimal number of genes, enzymes, ribosomes, tRNAs and low molecular weight compounds that are encapsulated within a synthetic compartment as in the case of lipid vesicles. The resulting construct, which is similar to living cells and displays minimal living properties (self-maintenance, self-reproduction and possibility to evolve) is generally designed on the basis of the minimal number of functions required and on the minimal complexity of the biochemical elements needed for its construction. Reproduced from [35] with permission of Elsevier.

The construction of minimal cells is carried out by following the "semi-synthetic approach"[4]: thanks to the self-organizing behavior of lipid molecules, spherical cell-like microcompartments - called lipid vesicles (liposomes) - form spontaneously in aqueous environments, encapsulating the molecular species present in solution (Figure 1).

Typically, nucleic acids, enzymes and other biomolecules are used, in order to reconstruct minimal versions of cellular functions such as nucleic acids replication, proteins synthesis or membrane growth. The ultimate goal of the semi-synthetic approach is the reconstruction of a cell-like system, capable of self-producing all its parts, and therefore able to grow and divide recursively. In addition to its potential relevance for biotechnology, this approach might also shed light on the biophysics and biochemistry related to the origin of early cells on Earth [4].

In the past, enzymatic reactions, such as oligonucleotide synthesis [5] or even PCR [6], were carried inside lipid vesicle. In 1999 it was reported the first micro-compartmentalized polypeptide synthesis [7], and soon after the first synthesis of a functional protein (the green fluorescent protein, GFP) [8]. To date, several reports deal with the fully understanding of the intra-vesicle protein synthesis, which is considered the keystone of the construction of semi-synthetic minimal cells (for a recent review, see [3]). In fact, the production of functional water-soluble or lipid-soluble [9] proteins paves the way to the reconstruction of the minimal number of cellular functions to achieve a full self-reproducing system.

### The encapsulation of solutes inside lipid vesicles

The key process underlying the construction of semi-synthetic minimal cells is the encapsulation (or "entrapment") of biomolecules in lipid vesicles. When such process occurs spontaneously, lipid microcompartments of several size and morphology, each containing different quantities of entrapped solutes are typically formed.

Due to the stochastic nature of this encapsulation process, the concentrations of the molecular species inside nanoscale (<1 µm of diameter) vesicles are not always similar to the external solution. In the case of a single solute, its intra-vesicle concentration can be lower or higher than the bulk one; in the case of multiple solutes, each species can be encapsulated with a different efficiency, creating a vast array of possibilities.

All this means that each individual vesicle behave as a unique microscopic bioreactor having a unique composition in terms of internalized components, which affects its performances in terms of effectiveness of internal reactions (the occurrence, or not, of a certain reaction, its rate and yield). There are clear evidences that populations of spontaneously formed vesicles, irrespective of their sizes, are heterogeneous in terms of individual performances (e.g., as revealed by the intra-vesicle protein production). Flow cytometry [10,11] and direct microscopic observations [12] have been used to monitor the time course of protein synthesis inside large (giant) vesicles. It is more difficult to perform similar studies in submicron vesicles, where it is expected, however, a higher diversity due to the enhancement of stochastic events at a smaller scale. Quite interestingly, two recent studies demonstrated that when 200 nm (diameter) lipid vesicles are prepared in presence of single macromolecular species, like ferritin [13] or ribosomes [14], the solute encapsulation does not follow the expected behavior. It resulted, instead, in a dramatic dichotomy between many "empty" vesicles and few "super-crowded" ones (having an internal solute concentration up to 60 times higher than the expected value). This reveals a marked non-random behavior of the entrapment process, such that the frequency distribution of the super-crowded liposomes seems to follow the Zipf-Mandlebrot law that is a *power-law *distribution. The volume dependency of this super-concentration effect seems to follow a power law too, resulting to be extremely marked as the vesicle diameter decreases.

These findings show that the issue of multi-solute encapsulation inside liposomes is a complex event that is far to be completely understood and that heavily affects the reactions occurring inside semi-synthetic minimal cells.

### Protein synthesis inside liposomes

As it has been mentioned, the technology for constructing semi-synthetic minimal cells is based on the integration of liposomes and cell-free technologies. Protein synthesis is considered one of the most relevant biological processes, and therefore it is currently studied in great detail.

Most of the work is carried out by employing a completely controllable protein production system, the PURESYSTEM™ (PS) [15]. It contains more than 80 macromolecular species (tRNAs included), and represents the minimal collection of biochemical components able to afford protein production from a coding DNA sequence. This feature is of fundamental importance considering the necessity to use known and controllable molecular components to reconstruct biological functions inside lipid micro-compartment, and moreover it is well-characterized in terms of its part, following the philosophy of synthetic biology.

Thanks to the encapsulation of all PS species (macromolecules and small molecules) inside lipid vesicles, it is possible to produce not only the GFP, but also functional enzymes as reported in several recent studies (see [3] for a review).

Clearly, the vesicle encapsulation of the PS should be viewed as a complex event, that is regulated by several solute-solute and solute-membrane interactions, by stochastic (and local) factors, and ultimately represents a realistic model of molecular self-organization at nanoscale range. As a result of this complex pattern, a vesicle population will be characterized by functional and structural heterogeneity that need to be studied without averaging out the inter-vesicle differences. In fact, the spreading of vesicle properties results in a kind of phenotypic diversity [16].

## Approach

### General strategy

Considering the relevance of protein synthesis in the construction of semi-synthetic minimal cells, and its potential new biotechnological applications [17], it is necessary to give a complete description of the protein production kinetics inside each lipid compartment. The reasons for this need are manifold. First, for progressing in the semi-synthetic approach to better design the construction of synthetic cells, we need to have a complete understanding of compartmentalized biochemical processes. Second, due to the difficulty of measuring the intra-vesicle composition in the case of complex mixtures, we need a tool for its evaluation, even if indirect. Third, despite the recent expansion of the field, not many studies have revealed the diversity of reaction kinetics inside vesicles and in particular in the case of the complex reaction that leads to protein synthesis.

An experimental approach uses liposomes of different size containing the DNA sequence encoding for a reporter molecule (the GFP, Green Fluorescent Protein) together with the PS, to detect and monitor the internal protein production over time. In the case of relatively large vesicles (above 0.5 micrometers in diameter) it is possible, at least in principle, to follow the course of the reaction in each vesicle thanks to light microscopy. Flow cytometry can also be used but only for following vesicles subpopulations. Large vesicles, however, due to their large size, show a minor degree in functional heterogeneity, that in turn derives from a more homogeneous internal composition.

In the case of submicron vesicles, where encapsulation anomalies are amplified (therefore representing a more interesting case) it is also possible to encapsulate the PS and produce a functional protein [18] revealing, at the same time, the "conundrum" of co-encapsulation. In this case, where multiple co-encapsulation events occur, the "anomalous entrapment" phenomenon (seen before for single molecular species [13,19]) acts by creating nanoscale liposomes containing hundreds of different molecules in a very small volume.

In both cases (large and small vesicles), it is difficult to measure directly the exact composition of each vesicle, putting severe limitations to the full understanding of the phenomenon of multiple co-entrapment. However, the GFP fluorescence signal emission allows the detection of the kinetics of protein production inside lipid vesicles, representing an important experimentally measurable parameter. As we have remarked, the different rate of protein production is due to the fact that each lipid vesicle encloses a different amount of each PS species, giving rise to a unique intra-vesicle biochemical composition.

With the purpose to obtain a tool which can correctly predict the kinetics of GFP production from different intra-vesicle PS compositions, we employed a computational approach to characterize in the detail the network of biochemical reactions of the entire transcription/translation process.

### Computational approach

In nanoscaled biochemical environment randomness and uncertainty cannot be described simply as an additive noise factor, but they represents the fundamental forces which drive the evolution of the system [20]. Nevertheless, to the best of our knowledge, a detailed stochastic description of *in lipo *protein synthesis has discussed in a very few papers [21].

To give a correct description of such small volume kinetics, the well-known Gillespie *Stochastic Simulation Algorithm *[22-24] was used, implemented in a simulation software.

A model for the translation module of the PS was published by our research team [21], comprising over 100 biochemical reactions with their kinetic coefficients; although this model is very complex, it was conceived to give a qualitative description of protein synthesis in different sized compartments, according to different entrapment models. As every first attempt, it contained some simplifications:

1) the presence of multiple elongating ribosomes on the same RNA molecule was not described;

2) protein production was conceived without taking into account aminoacids consumption;

3) the transcription process was not included;

These three problems are deeply intertwined, because the transcription and the translation processes are dynamically coupled in the PS: in the ribosomes bind to the Shine-Dalgarno sequence (the ribosomal binding site, or RBS) as soon as it is available, even if the complete RNA sequence has not been entirely produced yet. This means that the translation process begins as the RNA polymerase is still transcribing the RNA molecule, and the two processes (transcription and translation) cannot be treated as independent parallel processes.

Moreover, different ribosomes were detected as progressing along the same RNA molecule, and the same phenomenon was observed for transcription: multiple RNA polymerases can simultaneously transcribe the same DNA molecule.

A suitable *in silico *model for the PS must describe correctly all these interdependent processes.

By following the simulation strategy described in the "Materials and methods" section, we obtained a new improved *in silico *formalization of the PS that accounts for a detailed description of the single molecular reactions; aminoacids and nucleotides must be consumed in the right quantities to ultimately produce proteins and RNA.

The final goal of our approach is to investigate, by means of the obtained PS model, whether different initial concentrations of class of chemical species (DNA, enzymes, consumables) can give rise to significantly different kinetics of GFP production *in bulk*. Then we scaled the simulated liposomes down to nano-dimensions to assess whether these differences are detectable also in such very small vesicles. Our simulation approach allows us to forecast what GFP-production kinetics will be observed in dependence of different initial concentrations. Thus our model can be used in a reverse-engineering approach where the fluorescence data experimentally recorded will be used to infer the concentration of chemical species inside liposomes. This can reveal possible abnormalities in solutes entrapping mechanisms at nanoscale volumes.

## Results and discussion

### PS formal model

The obtained model consists of circa 280 different virtual species and 270 reactions, among which the vast majority represents dummy species or different molecular states used to obtain sequentiality through the strategy described in the Materials and Methods section. The full model is detailed in the sup. mat., here we describe the main simplifications.

Only GTP and ATP are included in the model, as the two fundamental energy resources used for peptide elongation (GTP), aminoacyl-tRNA charging (ATP) and transcription (both). The incorporation of also CTP and UTP requires the explicit declaration of additional ≈ 100 virtual species and even more biochemical reactions. Simulations experiments carried by adding CTP and UTP to the presented model output results undistinguishable from those of the simplified model (data not shown).

The forward motion of RNA polymerases and ribosomes is dependent by the availability of DNA/RNA sites; this formulation does not take into account whether the available sites are on the same DNA/RNA strand or not. This simplification is not affecting the consistency of the model, as the average number of elongating RNA polymerases per DNA strand remains the same using 1, 10 or 20 DNA molecules.

Amino acids, aminoacyl-tRNA synthetases, tRNAs, and Release Factors are modeled each as a single species, allowing a strong reduction of computational costs, which are notoriously high for SSA-based *in silico *experiments.

Despite these simplifications, the portrayed model avoids the use of simplified average macroscopic measures for the formalization of initiation, elongation or termination events. Moreover, this model accounts for the presence of ordered sequential events in the elongation steps, taking into account the steric repulsions between molecules and the correct sites occupancies.

The presence of multiple elongation events, dynamical coupling between Transcription and Translation and sequentially ordered motion of molecules were permitted in the model formulation itself, avoiding the addition of new reactions at fixed times which can jeopardize the stochastic description of the system [25].

### Overall consistency of the PS model

The validation of the proposed model has been obtained by comparing the RNA and protein production rates with available literature data, which show a range between 2.2 to 250 nt/s for the transcription process and a range between 0.03 to 15 aa/s [26-28] for translation. Extracted data for polymerases and ribosomes activities for the transcription/translation processes -19 nt/s and 4 aa/s, respectively- are consistent with experimental records.

### Varying the initial concentrations in large reaction volumes

Different biochemical compositions were tested *in silico*, using the standard concentrations of species in PS reported in [15], and varying the initial quantities of the 3 main classes of chemical species: DNA (*eGFP *sequence), enzymes (translation factors, ribosomes, polymerases, amino acyl-tRNA synthetases and energy recycling enzymes) or consumables (aminoacids, tRNAs, NTPs and other energy resources). They were lowered to 2/3 or 1/3 of their original value, for a total of 3^3^ = 27 combinations; each combination is defined by a series of 3 digits, accounting for DNA, enzymes or consumables concentrations; each digit contains a number which is 0,1 or 2, meaning respectively 1/3, 2/3 or 3/3 of their normal concentration. For example, "201" means 3/3 of DNA, 1/3 of enzymes and 2/3 of consumables.

Data for protein production over time was nicely fitted (R^2^>0.98) by sigmoid curves

$$y=\frac{GFP_{max}}{\begin{array}{c} 1+e^{-\left(\frac{x-x_{0.5GFP_{max}}}{b}\right)} \end{array}}$$

with parameters:

*GFP_max _*= maximum value of protein produced

*b *= maximum slope of the curve (a low value for b indicates an high steepness)

$$x_{0.5GFP_{max}}=\text{time value for y}=GFPmax/\text{2}$$

**Figure 2:**
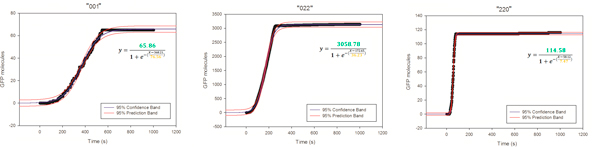
**Model fitting for simulated GFP production**. Protein production time courses averaged on 4 replicates (points) were fitted by 3-parameter sigmoid functions (red curves R2>0.98). Three different initial PS combinations are shown as examples: ("001") left, ("022") middle and ("220") right plot respectively.

For each initial biochemical combination we extracted the *GFP_max _*and *b *parameters, to compare the total GFP yield and its production rate for the different PS compositions (Figure 2).

### General dependencies by the initial conditions

**Figure 3:**
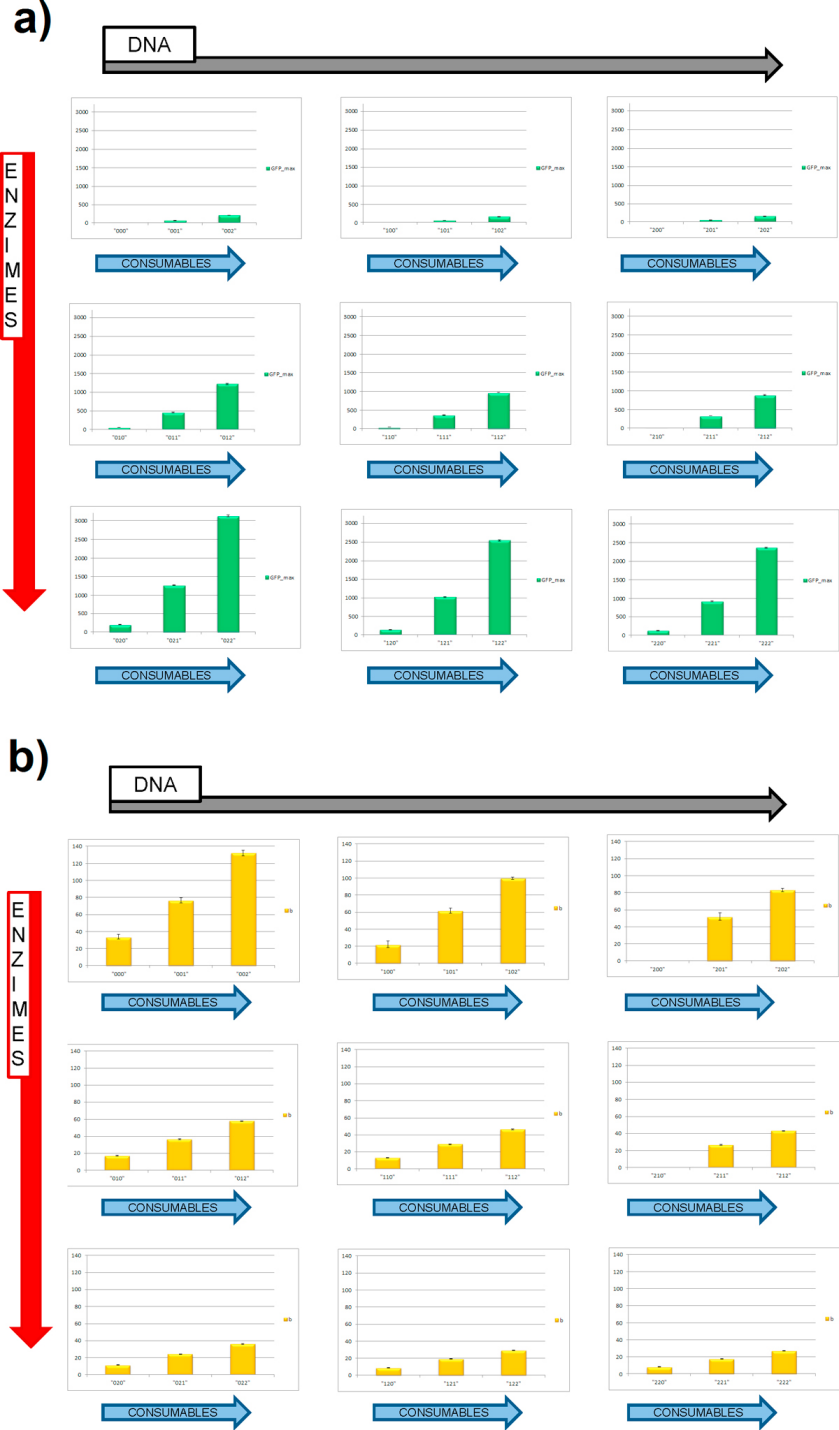
**Protein production kinetics for different PS compositions**. Parameters comparison for GFP production between different PS composition in a 10^-14 ^liters vesicle; a) total GFP yield (*GFP_max_*) and b) rate of protein production (*b*) are shown. Data comes from 4 replicates. Error bars refer to the standard error of the mean.

Figure 3 shows how the overall yield (*GFP_max_*) and kinetics (*b*) of GFP production change varying the initial amount of DNA, enzymes, or consumables in a 2.67µm-diameter vesicle (volume = 10^-14 ^L), with the purpose to study the general behavior of the system in presence of large number of molecules.

High DNA concentrations accelerates the overall protein production; however, the overall protein yield (*GFP_max_*) diminishes as the DNA amount increases; in fact, although rapid, protein production with high DNA concentrations stops at lower time values (see parameter $x_{0.5GFP_{max}}$in the Additional file 3). Simulations carried with a lower amount of enzymes concentrations resulted in a strong decrease of protein yield: a change from 3/3 to 2/3 in enzymes concentration determined a reduction in protein yield to circa 40% of the total; an additional reduction in enzymes concentration to a 1/3 of the original value led to a 5% of produced protein compared to normal conditions. In addition, very slow kinetics (high values for *b *parameter) are observed as the enzymes concentration decreases.

Input files with 1/3 of initial consumables amount yielded a maximum of only 200 protein molecules (internal concentration ≈ 33nM), revealing the role of energy resources as the fundamental factor which most affects the overall GFP production.

The highest protein production is afforded when DNA is low and enzyme and consumables are present in maximum quantity (sample "022"), reaching a total of approximately 3100 GFP molecules (final concentration ≈ 0.5 µM).

Trying to establish the exact role of DNA concentration in the protein production kinetics we performed stochastic simulations experiments using different lower DNA initial amounts.

As reported before, low DNA concentrations determine a slow kinetic of protein production: simulation performed using very low DNA amounts resulted in unfeasibly slow GFP productions. Furthermore, protein production encompasses several hours of time, and self-inactivating phenomena, which probably involve ribosomes inactivation, were experimentally observed after approximately 3 hours from the beginning of the experiment [26];

Thus, ad hoc simulations were performed to account for a self-inactivating reaction for ribosomes after circa 3 hours.

Data was fitted by sigmoid curves and the extracted parameters were compared as discussed before. Final protein yields are shown for different initial DNA amounts.

As DNA concentration decreases, the transcription process slows down, and GFP production kinetics are slower, but the total protein yield is increased to a total of over 9.500 GFP molecules (≈ 1.6 µM) when [DNA] = 15nM, a result which is comparable with experimental measures in GFP-expressing giant lipid vesicles of equal volume [10]. The non-linear dependence of total protein yield on the initial DNA concentration is the result of the balancing of two opposite phenomena: at very low initial DNA concentrations, the protein production goes slow, but almost all the chemical energy contained in the liposome can be used to produce the protein. On the other hand, when initial DNA concentration is high, the protein production is fast, but the energy consumption is even faster, making it impossible to obtain a larger protein yield (see the data in the Additional file 4).

**Figure 4:**
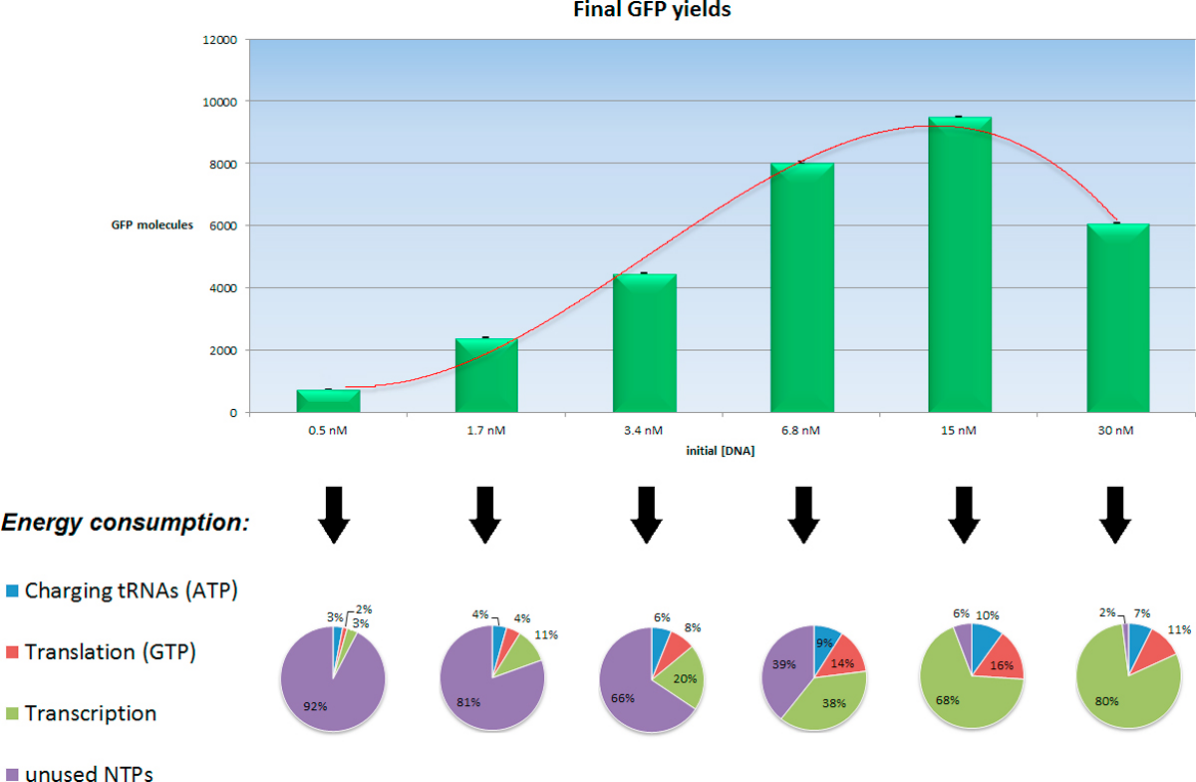
**Energy consumption varying the initial DNA concentration**. Maximum GFP yields for lower initial DNA concentrations. NTPs consumption is reported for each category, showing the role of the transcription process in accelerating protein production along with its high energy cost. Data from 10 replicates.

In fact, these results about the behavior of the PS (with the concentrations used in this work) showed how the competition between the ribosomes inactivation process and protein production speed determines a critical DNA concentration value, which delineates an optimal distribution of energy resources between the different processes (Figure 4).

### Energetic assessment of the PS

When using lower initial DNA concentrations (<100 nM) the competition effect between the transcription and translation processes for energy resources becomes a clearer phenomenon: Figure 4 illustrates the primal role of DNA in determining the overall GFP yield, and how scaling the initial amount of [DNA] results in a different energy consumption between the biochemical processes present in the PS reaction network.

When DNA concentration is high, transcription produces immediately many ribosomal binding sites and the translation initiation (which is one of the rate-limiting process of protein production) is more likely to occur, resulting in a more rapid protein production kinetic compared with inputs containing lower DNA concentrations, but at the same time, the transcription process consumes a large amount of nucleotides during the RNA elongation process.

Translation factors (IF1, Ef-Tu etc...) use GTP as energy donor, thus the lack of GTP molecules causes the translation process, and subsequently protein production, to stop. NDK (which is present in very low concentrations) can provide new GTP molecules from the ATP pool, but the additional GTP is consumed by transcription or in the intermediate translation steps, unlikely resulting in the formation of a significant number of new complete proteins. This simulation study allows us to assess two results. Firstly, the small changes in the initial concentrations of the PS chemical classes significantly influence the final GFP production and kinetics (a direct consequence of the non-linear behavior of the PS). Secondly, the initial DNA concentration can be deduced from the global GFP production, thus using the proposed model for a reverse engineering approach.

### At nanoscale range

The different protein yields for the different PS combinations were tested also in small volumes (10^-16 ^liters, corresponding to a vesicle with 575 nm of diameter); parameters were extracted after data fitting using the same procedure discussed before.

**Figure 5:**
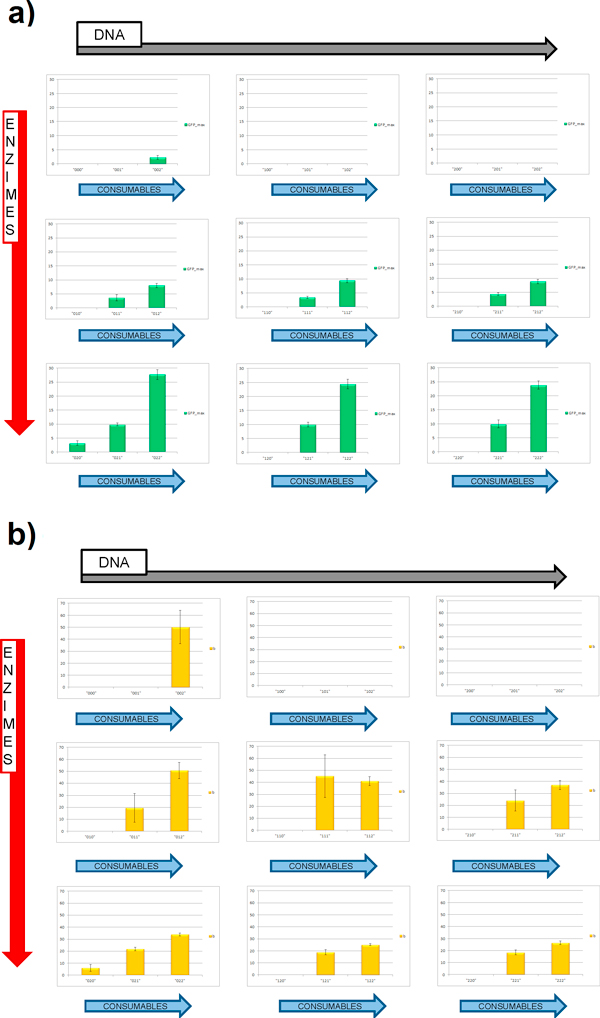
**Protein production kinetics for different PS compositions**. Parameters comparison for GFP production between different PS composition in a 10^-16 ^liters vesicle; a) total GFP yield and b) rate of protein production are shown. Data comes from 8 replicates. Different combinations are not able to produce GFP. Error bars refer to the standard error of the mean.

The obtained results (Figure 5) showed a similar distribution of the GFP production efficiencies, reaching a maximum of approximately 26 proteins (final protein concentration ≈ 0.44 µM) for the "022" sample, as seen also for simulations in higher volumes.

Many combinations resulted in negligible GFP production, indicating the importance in lower volume of an optimal internal biochemical composition.

The general trend for protein yield/kinetics and its dependencies by DNA/enzymes/consumables concentrations is again predicted in small volumes, showing the behavior of the PS when no anomalous entrapment phenomena are supposed. In all the 27 different initial conditions, we can observe the relative low importance of the DNA quantity, probably because it is anyhow relatively high with respect to the initial concentration of enzymes and consumables. This situation is a direct consequence of the high discretization level: to have a fully functional liposome, at least one complete DNA molecule should be entrapped. This may alter the exact proportionality with the initial concentration in bulk. We finally observe that the combination "x22" (where × means that the DNA initial concentration does not affect the final result) shows the maximum estimated protein production. To suggest an experimental test, we recommend to compare the "200" and the "022" samples, in order to have the maximum possible resolution for the differences in GFP production, between the two initial conditions.

Concerning the kinetics studies, we confirm that the two optimal experimental candidates are the "200" and "022" samples, but we observe that the kinetic parameter is critically dependent on the initial enzymes concentration, that, at these small volumes, can show very high random fluctuations (data are stored in the Additional file 5).

We remark that also in small volumes, the small changes in initial concentrations lead to significantly different final statuses, thus supporting the proposed experimental design.

## Conclusions

With this work we have conquered a methodological goal, finding a way to describe sequential processes in a concurrent-only stochastic simulation environment. Thanks to this we have given the most detailed description of the PS ever proposed. We have then explored different initial concentration and forecasted the kinetics of protein synthesis for large and small volume liposomes. Within the small volumes, we isolated extreme final values of GFP-production in order to select candidate of experimentally distinguishable cases. We present our platform as a reverse-engineering tool to be used to analyze future experimental data.

## Materials and methods

### QDC and its input language

The formal specification of the PS reaction network was assessed using a stochastic simulation software previously built by our team, QDC (Quick Direct-method Controlled), which is based on the Direct Method version of the Gillespie Stochastic Simulation Algorithm [29]. QDC's input language is simple and intuitive, and easily recalls the standard notation for biochemical reactions.

**Figure 6:**
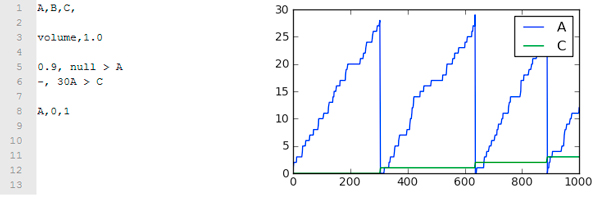
**QDC's syntax example**. Here it is an example of an immediate reactions; the A molecule is continuously added by the uptake reaction 0.9 null > A; the immediate reaction is identified by the hyphen sign ("-") that is present instead of a kinetic coefficient, and acts by producing one C molecule every 30 A molecules; notice the resulting oscillatory behavior of the A specie.

QDC has been designed to simulate experiments performed on metabolic networks, where the operator can exert three different control action on the metabolic network: 1) add or remove molecules at a given time; 2) change the propensity of a reaction at a given time; 3) simulate all-or-nothing reactions (called "*immediate reactions*" in QDC's language), where the chemical reaction is executed immediately after the stoichiometry of the left part of the equation is satisfied (Figure 6).

*Immediate *reactions are not standard biochemical events that occur accordingly with a kinetic law, but they rather represent logical statements, which were introduced to allow the description of complex condition with possibly many chemical species interacting.

These features of QDC allowed us to build a detailed model of the PS reaction network. Biochemical reactions with their relative constants from the previous model [21] were updated, when possible, using data from literature and the BioNumbers [30] database.

The transcription reactions were modeled using data from published material [31]; the translational core model was updated using a more detailed kinetic description [32].

### In silico PURESYSTEM model

The first result obtained in this work has been a detailed formal description of the PS: every molecular component was explicitly inserted in the model together with the known biochemical reactions between the different reagents, as described in the previous section.

The same model simplifications present in the previous work [21] were introduced to create a suitable transcription/translation formulation.

**Figure 7:**
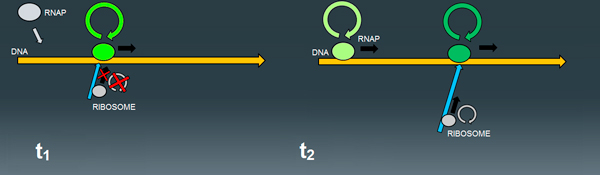
**Transcription/translation process in the PS**. (t_1_) The ribosome cannot incorporate amino acids and subsequently move forward if the polymerase (RNAP) has not yet produced a sufficiently long RNA sequence; (t_2_) as long as transcription continues, new nucleotides are incorporated, the RNA molecule is elongated, and the ribosome can continue the translation process and progress along the RNA strand, while a new polymerase bind to DNA and begins to produce a new RNA molecule, by keeping a certain distance from the other elongating polymerase due to steric repulsions.

However, as pointed in the previous section, a formal description of interdependent elongation events transcends the standard SSA specifications, which depicts the system evolution according to propensities rules only proportional to the number of reacting molecules, without accounting for the presence of sequential processes or topological constraints (Figure 7).

**Figure 8:**
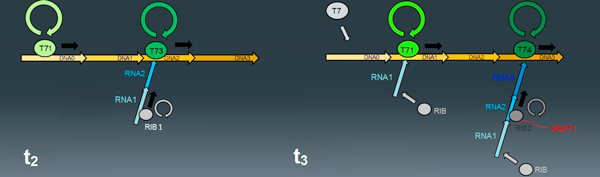
**Incorporation of different molecular states**. (t2) A newly-bond polymerase (T71) can advance to the next DNA sequence (DNA1) which is free, and the ribosome (RIB1) can begin to incorporate amino acids and move forward, because the RNA sequence has been extended and the adjacent site (RNA2) is free; in the next stage (t3), polymerases and ribosomes have advanced, consequently releasing the site they were previously occupying; accordingly, a new polymerase (T7) and a new ribosome (RIB) can bind to their respective target molecules, and start the elongation processes.

To coherently describe the proper distance between elongating molecules and, subsequently, a correct occupancy of RNA and DNA sequences, all the species involved in the elongation events (DNA, RNA, polymerases and ribosomes) were split into multiple entities representing different molecular *states *(Figure 8).

The divisions in multiple molecular states allows a coherent organization of site occupancies and dynamic motion, but the transition events describing the forward motion of ribosomes and polymerases are two processes which act in a sequential fashion.

The solution to this problem was assessed using logical statements written in form of reactions present in the QDC syntax, the aforementioned immediate reactions, which were massively used in this new model to regulate the forward motion of polymerases and ribosomes, accounting for correct spacing and dynamical coupling.

### Modeling strategy

The DNA sequence encoding for GFP was divided, according its length, into different multiple species, each representing a 80 bp sequence; the polymerization process is divided into different reactions, describing a second-order reaction for nucleotide binding, and a first-order reaction for nucleotides incorporation which returns the polymerase molecule (which can bind to another nucleotide, see the blue braces) and a "dummy" product, that allows to track the number of nucleotides incorporated in the RNA molecule (the term *dummy *comes from computer science language, where dummy variables are arbitrary chosen variable employed for temporary purposes); the immediate reactions determine the transition to the next step, ensuring the following conditions: a) an adjacent DNA site is available, b) a correct number of nucleotides has been added to the RNA sequence, c) the corresponding RNA sequence is produced, d) the previously occupied DNA site is released. Here it is an example for transcription process at the fourth step (GTP and ATP are considered):

1000000, **T7ELGT4 **+ GTP > T7pregEL4

28, T7pregEL4 > T7ELAT4 + Pi + **gtr4**

1000000, T7ELAT4 + ATP > T7preaEL4

28, T7preaEL4 >**T7ELGT4 **+ Pi + **atr4**

-, 20 **gtr4 **+ 20 **atr4 **+ T7ELGT4 + *DNA5 *> T7ELGT5 + RNA4 + *DNA3*

This reaction "box" was duplicated different times ensuring the correct succession of molecular states; when only one DNA molecule is available, the polymerases advance one by one, separated by at least one DNA site between each other (elongating RNA polymerases are separated each other by at least 80 bp [33]). The same strategy was used to describe the translation reactions.

100000000, **eR3 **+ EFaRGTP > EXpre3

79, EXpre3 > eR3 + EFaRGTP

207, EXpre3 > EX3

3.45, EX3 > EXpre3

100, EX3 > EX3GDP + Pi

638, EX3GDP > eRa3GDP

15, eRa3GDP > eRa3 + EFtuGDP

20, eRa3 > eRa3tRNA

150000000, eRa3tRNA + EFgGTP > EXbpre3

140, EXbpre3 > eRa3tRNA + EFgGTP

250, EXbpre3 > EXb3 + EFg + GDP +Pi

20, EXb3 >**eR3 **+ tRNA + **TRANSL3**

-, 27 **TRANSL3 **+ RNA4 + *PEPT2 *+ **eR3 **> eR4 + *PEPT3 *+ RNA2

An elongating ribosome (eR2) binds the complex which carry the aminoacid (EFaRGTP), after which moves to the next codon, aided by the elongation factor EFg charged with GTP (EFgGTP); this translocation reaction yield ad additional product which is used to regulate the progression to the next state.

After a fixed number of translocation steps (the minimal space between two elongating ribosomes is, as for polymerases, 80 nt ≈ 27 codons [34]) an immediate reaction occurs in a similar fashion as seen for transcription: 1) the correct amount of aminoacids are incorporated, and thus consumed; 2) the next free RNA site is occupied and 3) the previous one is therefore liberated; 4) an entity named PEPT is also produced, allowing to calculate the length of the peptide sequence produced so far: for example, if 4 species named PEPT3 are present in a certain time of the simulation, this means that there are 4 peptides, still bound to the ribosomes, with a length spanning from 27 × 3 = 81 to (27 × 4)-1 = 107 aminoacids. Additional file 1 contains a legend of all the chemical species declared in our *in silico *PS, while Additional file 2 contains the complete input file for simulating by QDC the PS entrapped in a 10^-16 ^L liposome.

## List of abbreviations used

PS: PURESYSTEM™; POPC: 1-palmitoyl-2-oleoyl-*sn*-glycero-3-phosphatidylcholine; NDK: Nucleotide Diphosphate Kinase; GFP: Green Fluorescent Protein; ATP: Adenosine Triphosphate; GTP: Guanosine Triphosphate.

## Competing interests

The authors declare that they have no competing interests.

## Supplementary Material

Additional file 1 **A text file with the legend of all the names used for chemical species in the input file**. Click here for file

Additional file 2 The input file for a liposome of 10**^-16 ^**L; this is readable by QDC simulator. Click here for file

Additional file 3 **A MS-Excel file containing all the results of the simulations performed for large-volume liposomes**. Click here for file

Additional file 4 **A MS-Excel file containing all the results of the simulations performed for very low initial DNA concentrations**. Click here for file

Additional file 5 **A MS-Excel file containing all the results of the simulations performed for small-volume liposomes**. Click here for file

## Declarations

The Authors declare that this paper is part of the supplement dedicated to BITS2012 conference. The funds covering the publication fee have been collected from research funds assigned by Authors' Departments and the MIUR Project PRIN 2008FY7RJ4.

This article has been published as part of *BMC Bioinformatics *Volume 14 Supplement 7, 2013: **Italian Society of Bioinformatics (BITS): Annual Meeting 2012**. The full contents of the supplement are available online at http://www.biomedcentral.com/bmcbioinformatics/supplements/14/S7
